# Left Atrial Function and Strain Assessed by Three‐Dimensional Echocardiography as Factors Associated With Atrial Fibrillation Recurrence After Pulmonary Vein Ablation

**DOI:** 10.1111/jce.16757

**Published:** 2025-07-01

**Authors:** Andrea P. Maldonado‐Tenesaca, Elias N. Andrade Cuellar, Froylan D. Martínez‐Sánchez, Rocio Aceves‐Millan, Juan Carlos Solis‐Gómez, Jose J. I. Y. Zamora‐Diaz, Martin R. Cedillo‐Urbina, Carlos H. Ixcamparij‐Rosales, Rogelio Robledo‐Nolasco

**Affiliations:** ^1^ Echocardiography Laboratory, Centro Médico Nacional 20 de Noviembre ISSSTE Mexico City Mexico; ^2^ Cardiac Electrophysiology, Centro Médico Nacional 20 de Noviembre ISSSTE Mexico City Mexico; ^3^ Department of Cardiology Centro Médico Nacional 20 de Noviembre ISSSTE Mexico City Mexico; ^4^ Facultad de Medicina Universidad Nacional Autonoma de Mexico Mexico City Mexico; ^5^ Department of Internal Medicine Hospital General “Dr. Manuel Gea González” Mexico City Mexico

**Keywords:** atrial fibrillation, atrial strain, left atrium, pulmonary vein ablation, three‐dimensional echocardiography

## Abstract

**Introduction:**

Recurrence of atrial fibrillation (AF) after pulmonary vein isolation (PVI) remains a major clinical challenge. Left atrial (LA) remodeling contributes to recurrence, but its predictive value is still under investigation. This study aimed to assess LA morphology, function, and strain using three‐dimensional (3D) echocardiography and determine their association with AF recurrence.

**Methods and Results:**

This prospective, single‐center study included 119 patients with paroxysmal or persistent AF who underwent radiofrequency or cryoballoon ablation. Six months after the procedure, an echocardiographic assessment was performed, evaluating LA volumes, reservoir, conduit, booster pump function, global longitudinal strain (GLS), and LA ejection fraction (LAEF).

AF recurrence occurred in 26.1% of patients. Patients with recurrence had larger LA dimensions (*p* < 0.01) and reduced GLS, LAEF, and peak atrial longitudinal strain (PALS) (*p* < 0.001). Multivariate analysis identified GLS > −13.5% (OR = 3.495, *p* < 0.001) and LAEF < 27.5% (OR = 0.857, *p* < 0.001) as independent predictors of recurrence. ROC analysis confirmed their strong discriminatory ability (AUC: 0.941 and 0.812, respectively).

**Conclusion:**

LA remodeling and dysfunction are key predictors of AF recurrence after PVI. Reduced GLS and LAEF and increased LA size were independently associated with recurrence, supporting their role in risk stratification and personalized management. Incorporating 3D echocardiographic and strain‐based analysis may enhance postablation surveillance and therapeutic decision‐making.

## 1 Introduction

Atrial fibrillation (AF) is the most prevalent cardiac arrhythmia worldwide, affecting approximately 4% of adults and over 5 million people globally [1, 2, 3]. This condition, characterized by rapid and irregular atrial activation, is associated with a significantly increased risk of thromboembolic events, heart failure, and reduced quality of life while also posing a substantial burden on healthcare systems [3, 4]. From a pathophysiological perspective, AF induces electrical, functional, and structural remodeling of the left atrium (LA), including atrial dilation and fibrosis, perpetuating the arrhythmic substrate and complicating clinical management [1, 2, 3, 5].

Pulmonary vein isolation (PVI) via cryoballoon or radiofrequency ablation is a cornerstone therapy for rhythm control in patients with symptomatic AF refractory to antiarrhythmic drugs [6, 7]. While landmark trials such as EAST‐AFNET4 and CABANA have demonstrated that PVI alleviates symptoms, reduces hospitalizations, and improves survival in selected patients, postprocedural recurrence rates remain high, reaching up to 50% in persistent AF and 30% in paroxysmal AF [8, 9, 10]. This outcome variability highlights the importance of accurate predictive markers to enhance patient selection and treatment approaches [2, 11, 12, 13].

In this context, left atrial volume and phasic function (reservoir and conduit), along with atrial strain parameters assessed through two‐dimensional, speckle‐tracking echocardiography, have emerged as essential tools for characterizing the atrial substrate and predicting AF recurrence [14]. This study comprehensively analyzes LA echocardiographic parameters in patients undergoing pulmonary vein isolation, establishing associations between these measurements and AF recurrence to contribute to developing personalized strategies that improve clinical outcomes.

## 2 Methods

### 2.1 Study Population and Protocol

This single‐center cross‐sectional study included 119 adult patients ( ≥ 18 years) with symptomatic AF, either paroxysmal or persistent, who had PVI. Patients with moderate or severe valvular disease, prosthetic heart valves, or inadequate image quality for proper echocardiographic assessment were excluded. All participants provided written informed consent, and the institutional ethics committee (05‐217‐2024) approved the protocol by the Declaration of Helsinki.

### 2.2 Ablation Protocol

The catheter mapping and ablation approach followed standard practice. Anticoagulants were not discontinued before the procedure. Patients were intubated and mechanically ventilated under deep sedation.

For radiofrequency ablation (RFA), electroanatomical mapping was performed in sinus rhythm; if the patient was in AF during the procedure, electrical cardioversion was performed beforehand. Two femoral vein punctures were made: one was used to introduce an 8 Fr or 10 Fr sheath, while the other allowed the advancement of a Preface sheath with a Brockenbrough needle for transseptal puncture.

With the support of a deflectable sheath (CARTO VIZIGO®), electroanatomical mapping was conducted using a Pentaray or Octaray catheter (Biosense Webster Inc.) and the CARTO 3® platform with the CONFIDENSE™ system. Bipolar mapping was performed within a voltage range of 0.1–0.5 mV. Ablation was carried out using an irrigated catheter with contact force sensing (Smart Touch) at high‐power settings (50 W for the anterior wall and 40 W for the posterior wall), guided by an ablation index of 500 in the anterior wall and 400 in the posterior wall. Heparin was administered every 20 min to maintain an activated clotting time (ACT) between 250 and 350 s.

For cryoballoon ablation (CBA), a second‐generation cryoballoon (Arctic Front Advance, Medtronic) was used, ensuring at least two successful applications per pulmonary vein.

In patients with persistent AF, roofline ablation and ablation of fractionated atrial electrograms were performed when using radiofrequency energy. The primary goal of the procedure was to achieve a bidirectional conduction block in all pulmonary veins. Among the 52 patients with persistent atrial fibrillation, additional substrate modification (including roofline ablation and ablation of fractionated atrial electrograms) was performed in 46 patients (88.5%).

### 2.3 Clinical Follow‐Up

After ablation, all patients continued oral anticoagulation therapy. Antiarrhythmic treatment was adjusted according to the type of AF: beta‐blockers were prescribed for paroxysmal AF, while amiodarone was used for persistent AF, both maintained for 3 months. Antiarrhythmic therapy was discontinued based on clinical evolution (presence or absence of arrhythmia recurrence).

Clinical follow‐up included regular outpatient evaluations at 1, 3, and 6 months, supplemented by 24‐h Holter monitoring for arrhythmia detection at 3 and 6 months. AF recurrence was defined as any documented atrial tachyarrhythmia (clinically or via 24‐h Holter monitoring) lasting > 30 s beyond 12 weeks after pulmonary vein isolation.

### 2.4 Echocardiographic Evaluation

All patients underwent two‐dimensional (2D) and three‐dimensional (3D) transthoracic echocardiography (TTE) 6 months after PVI using a high‐end imaging system (GE E95 with a 4Vc‐D transducer). LA dimensions were assessed, including anteroposterior, longitudinal, and transverse diameters, and maximum LA volume indexed to body surface area.

**Figure 1:**
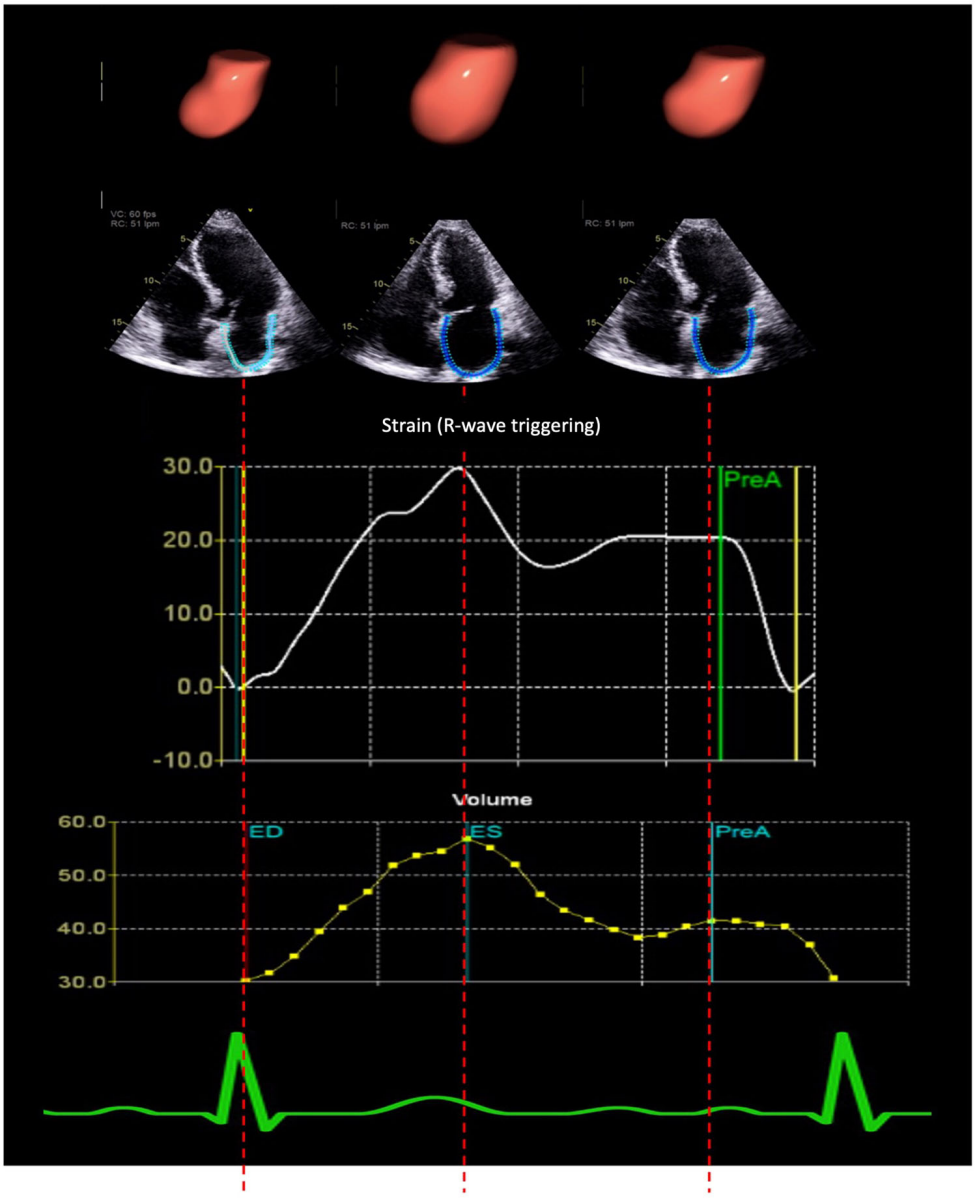
Left atrial volume and strain analysis. Left atrial (LA) volume and strain analysis using echocardiographic time‐volume and strain curves. The top row shows three‐dimensional (3D) reconstructions of the LA at different phases of the cardiac cycle: end‐diastole (ED), end‐systole (ES), and pre‐atrial contraction (PreA). The middle row displays two‐dimensional (2D) echocardiographic images with automated LA border detection at corresponding time points. The bottom row presents time‐strain and time‐volume curves, illustrating peak atrial longitudinal strain (PALS) at end‐ventricular systole and peak atrial contraction strain (PACS) before atrial contraction, along with LA phasic volumes. An ECG tracing is included for phase alignment. These measurements assess LA reservoir, conduit, and booster pump function, contributing to understanding LA remodeling and its role in atrial fibrillation recurrence.

**Figure 2:**
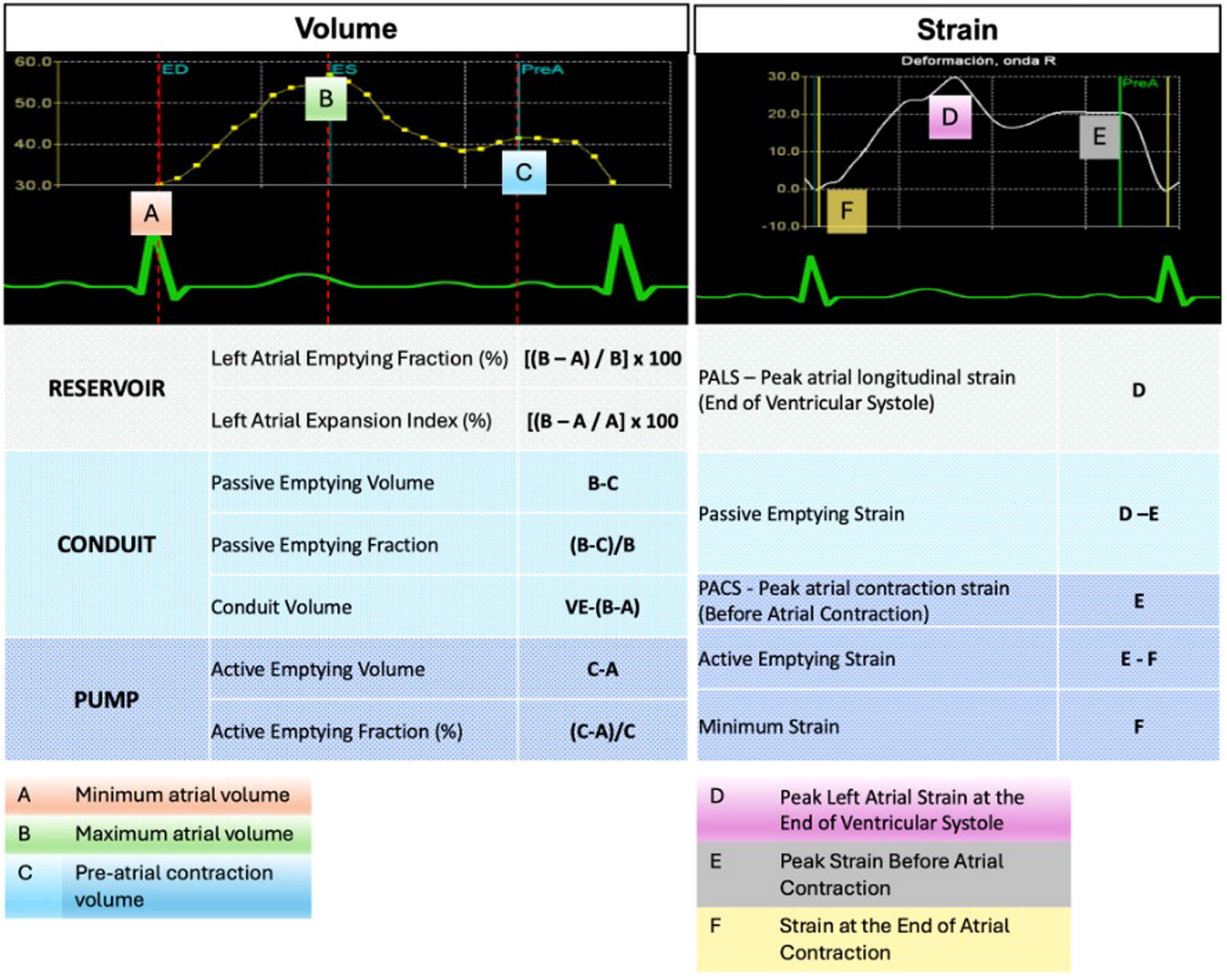
Left atrial phasic function and strain analysis using time‐volume and time‐strain curves. Left atrial (LA) phasic function and strain assessment using time‐volume and time‐strain curves. The left panel illustrates the LA reservoir, conduit, and pump function, with volume‐derived indices such as left atrial emptying fraction (LAEF) and left atrial expansion index (LAEI). The right panel shows the strain‐based evaluation, highlighting peak atrial longitudinal strain (PALS) at end‐ventricular systole, peak atrial contraction strain (PACS) before atrial contraction, and minimum strain at the end of atrial contraction. These indices comprehensively characterize LA function and remodeling, which are key predictors of atrial fibrillation recurrence.

Phasic LA volumes were obtained from time‐volume curves, and LA functional indices were calculated using previously validated formulas, as illustrated in Figure 1. Additionally, strain‐based parameters, including peak atrial longitudinal strain (PALS) and peak atrial contraction strain (PACS), were analyzed using time‐strain curves (Figure 2).

A full‐volume 3D data set of the LA was acquired from an apical four‐chamber view (Video 1). Multiplanar reconstruction defined the mitral annulus from a single reference point in the apical four‐chamber, two‐chamber, and three‐chamber views. Endocardial borders were manually adjusted at end‐diastole and end‐systole, followed by the GE E95 system automatic contour detection, which generated time‐volume curves (Videos 1 and 2).

Finally, additional parameters were assessed, including left ventricular ejection fraction (LVEF) using the biplane Simpson method, E/e′ ratio, pulmonary artery systolic pressure (PASP), and the LA stiffness index. All measurements were performed by an experienced echocardiographer blinded to clinical outcomes and were averaged from three cardiac cycles in sinus rhythm or five cycles in patients with AF (Video 3).

### 2.5 Statistical Analysis

All statistical analyses were performed using SPSS version 25 (IBM Corp., Armonk, NY, USA). Depending on the data distribution, continuous variables were expressed as mean ± standard deviation or median with an interquartile range, and they were assessed using the Kolmogorov–Smirnov test. Categorical variables were presented as absolute frequencies and percentages.

Comparisons between patients with and without AF recurrence were performed using the Student's t‐test or Mann–Whitney *U* test for continuous variables and the chi‐square test or Fisher's exact test for categorical variables, as appropriate. Spearman correlation analysis was used for non‐parametric variables to assess relationships between echocardiographic parameters and AF recurrence.

A univariate logistic regression analysis was conducted to identify potential predictors of AF recurrence. A multivariate logistic regression model included variables with *p* < 0.10 in univariate analysis, adjusting for clinically relevant covariates. Results were reported as odds ratios (OR) with 95% confidence intervals.

A receiver operating characteristic (ROC) curve analysis was performed to determine the predictive value of significant echocardiographic parameters. The area under the curve (AUC) was used to assess discriminatory ability, with optimal cutoff values identified using the Youden index. A *p*‐value < 0.05 was considered statistically significant for all tests.

A post hoc power analysis was conducted using G*Power version 3.1.9.7 to evaluate the adequacy of the sample size. Based on an observed odds ratio of 3.495 for GLS > –13.5%, a recurrence probability of 20% among exposed patients, a significance level (*α*) of 0.05, and *R*² = 0.1, the calculated statistical power for the logistic regression model was 99.8%. This confirms the robustness of the study to detect a significant association with high confidence.

**Video 1:** Left atrial strain curves.

**Video 2:** Left atrial volume curve.

**Video 3:** Full‐volume acquisition of the left atrium.

## 3 Results

### 3.1 Clinical and Demographic Data

The study included 119 patients with symptomatic AF who underwent PVI. The mean age was 65.8 ± 12.4 years; most patients were male (78.2%). The mean body mass index (BMI) was 27.64 ± 5.81 kg/m^2^, and the mean body surface area (BSA) was 1.88 ± 0.27 m^2^. Regarding comorbidities, 76.5% had hypertension, 25.2% had diabetes, 14.3% had a history of stroke, 33.6% had heart failure, and 47.9% had coronary artery disease. AF type distribution showed that 56.3% of patients had paroxysmal AF, while 43.7% had persistent AF. Regarding the procedural method, 68.9% of patients received radiofrequency ablation, while 31.1% opted for cryoballoon treatment. The overall AF recurrence rate was 26.1%, with 31 patients experiencing AF recurrence after PVI. Table 1 presents patients' clinical and demographic information categorized by their recurrence status. In the subgroup of 46 patients who received additional substrate modification, arrhythmia recurrence occurred in 18 patients (39.1%). Among them, 14 (77.8%) experienced recurrent atrial fibrillation and 4 (22.2%) had organized atrial tachycardia.

**Table 1:** Clinical and demographic by recurrence status.

Variable	Recurrence: No (*n* = 88)	Recurrence: Yes (*n* = 31)	*p* value
Type of AF *n* (%)			<0.001
Paroxysmal	60 (68.2)	7 (22.6)	
Persistent	28 (31.8)	24 (77.4)	
Sex *n* (%)			<0.001
Male	62 (70.5)	31 (100.0)	
Female	26 (29.5)	0 (0.0)	
Age (years)	65.25 ± 13.55	67.35 ± 8.26	0.419
BSA (m^2^)	1.82 ± 0.22	2.06 ± 0.31	<0.001
BMI (kg/m^2^)	25.82 ± 5.10	32.82 ± 4.44	<0.001
Hypertension *n* (%)			0.329
	65 (73.9)	26 (83.9)	
Diabetes *n* (%)			<0.001
	14 (15.9)	16 (51.6)	
Stroke *n* (%)			0.004
	8 (9.1)	9 (29.0)	
HF *n* (%)			0.007
	23 (26.1)	17 (54.8)	
Ischemic heart disease *n* (%)			0.012
	36 (40.9)	21 (67.7)	
Procedure *n* (%)			0.825
Cryoablation	28 (31.8)	9 (29.0)	
RF Ablation	60 (68.2)	22 (71.0)	

*Note: p* value: Chi‐square test for categorical variables and Student's T‐test for continuous parametric variables.

Abbreviations: AF, atrial fibrillation; BSA, body surface area; BMI, body mass index; HF, heart failure.

### 3.2 Echocardiographic Parameters

The median LA maximum volume (LAmax) was 52.00 (43–70) mL, whereas the median minimum LA volume (LAVmin) was 35 (ranging from 24 to 59) mL. The pre‐atrial contraction volume (Pre‐LAV) measured 45 (35–63) mL. The LA ejection fraction (LAEF) had a median value of 30% (26%–40%). Regarding strain parameters, the median PALS was 10% (7%–15%), while the median PACS was −7% (−10% to −5%). The median global longitudinal strain (GLS) was −14% (−17% to −13%).

The LA stiffness index had a median value of 0.81 (0.57–1.65), and the E/e′ ratio was 10 (6–12). Left ventricular (LV) function was assessed using the biplane Simpson method, with a median LVEF of 52% (45%–56%). The median left ventricular end‐diastolic volume (LVEDV) was 91 (70–118) mL, and the median left ventricular end‐systolic volume (LVESV) was 40 (33–52) mL. The PASP had a median value of 31 (28–39) mmHg.

Patients with AF recurrence exhibited significantly larger LA dimensions, including increased anteroposterior, longitudinal, and transverse diameters, and larger LA areas in both the four‐chamber and two‐chamber views (Table 2). In addition, LVEF was significantly lower in patients with AF recurrence.

**Table 2:** Left atrial (LA) and left ventricular (LV) morphology and hemodynamics.

Variable	Recurrence: No (*n* = 88)	Recurrence: Yes (*n* = 31)	*p* value	Spearman correlation	*p* value
LA morphology					
Anteroposterior diameter (mm)	41 (38, 44)	51 (46, 62)	< 0.001	0.449	< 0.001
Longitudinal diameter (mm)	55 (49, 60)	62 (61, 77)	< 0.001	0.425	< 0.001
Transverse diameter (mm)	41 (38.50, 48)	49. (48, 51)	< 0.001	0.321	< 0.001
LA area (4‐chamber view) (cm^2^)	19 (16, 23)	24 (24, 34)	< 0.001	0.349	< 0.001
LA area (2‐chamber view) (cm^2^)	19 (18,24)	28 (21, 35)	< 0.001	0.493	< 0.001
LV function					
LVEF (%)	55 (49, 57)	43 (36, 45)	< 0.001	−0.665	< 0.001
E/e′ ratio	9 (6, 11)	12 (6, 16)	0.010	0.238	0.009
LV end‐diastolic volume (mL)	91 (71, 104)	115 (48, 118)	0.528	0.058	0.531
LV end‐systolic volume (mL)	37 (33, 47)	65 (31, 65)	0.047	0.183	0.046
Global longitudinal strain (%)	−16.0 (−17.0, −14.0)	−9.7 (−13.0, −7.0)	< 0.001	0.679	< 0.001
Pulmonary artery systolic pressure (mmHg)	31.0 (28.5, 37.5)	41.0 (25.0, 45.0)	0.311	0.093	0.313

*Note: p* value: Mann–Whitney *U* for comparisons between both groups; Separman's correlation for nonparametric variables.

Abbreviation: LVEF, left ventricular ejection fraction.

Regarding LA function, patients with recurrence had higher maximum, minimum, and precontraction LA volumes (Table 3). Moreover, LA ejection fraction, PALS, LA emptying fraction, and LA expansion index were significantly reduced. While passive emptying volume was higher in patients with recurrence, differences in conduit function and booster pump parameters were not statistically significant.

**Table 3:** Left atrial (LA) function, reservoir, conduit, and booster pump.

Variable	Recurrence: No (*n* = 88)	Recurrence: Yes (*n* = 31)	*p* value	Spearman correlation	*p* value
Phasic LA function					
Maximum LA volume (mL)	51(42, 65)	64 (51, 74)	0.003	0.277	0.002
Minimum LA volume (mL)	30 (22, 58)	47 (37, 61)	< 0.001	0.340	< 0.001
Pre‐contraction LA volume (mL)	42 (33, 61)	59 (39, 66)	0.006	0.251	0.006
LA ejection fraction (%)	37 (29, 47)	26.00 (18, 27)	< 0.001	−0.475	< 0.001
Reservoir					
Peak atrial strain at the end of ventricular systole (%)	13 (7, 19)	8 (6, 10)	< 0.001	−0.377	< 0.001
LA emptying fraction (%)	36.8 (28.7, 47.4)	26.6 (17.6, 29.2)	< 0.001	−0.363	< 0.001
LA expansion index (%)	58 (40, 90)	36 (21, 41)	< 0.001	−0.363	< 0.001
Conduit					
Passive emptying volume (mL)	7 (3, 15)	8 (8, 17)	0.005	0.259	0.004
Passive emptying fraction (%)	15.38 (5.97, 23.91)	10.81 (10.81, 23.53)	0.716	0.033	0.718
Conduit volume	25 (17, 39)	36 (4, 36)	0.278	−0.100	0.280
Passive emptying strain	19 (12, 26)	13 (11, 22)	0.235	−0.109	0.236
Booster pump					
Peak atrial strain before atrial contraction (%)	−6 (−7, −5)	−7 (−5, −7)	0.415	−0.075	0.417

*Note: p* value: Mann–Whitney *U* for comparisons between both groups; Separman's correlation for nonparametric variables.

### 3.3 Multivariate Analysis Results

#### 3.3.1 LA and LV Morphology and Hemodynamics

Multivariate logistic regression analysis revealed that larger LA dimensions were independently associated with a higher risk of AF recurrence (Table 4). Specifically, anteroposterior, longitudinal, and transverse LA diameters and LA areas in the four‐chamber and two‐chamber views remained significant predictors (*p* < 0.01 for all). Among these, the two‐chamber LA area showed the strongest association (OR = 1.307, 95% CI: 1.174–1.454, *p* < 0.001). (Table 4)

**Table 4:** Logistic regression analysis for left atrial (LA) and left ventricular (LV) morphology and hemodynamics.

Variable	Univariate	Wald	*p* value	Multivariate	Wald	*p* value
LA morphology						
Anteroposterior diameter (mm)	1.110 (1.057–1.165)	17.768	< 0.001	1.175 (1.095–1.261)	20.031	< 0.001
Longitudinal diameter (mm)	1.076 (1.034–1.120)	13.107	< 0.001	1.094 (1.046–1.144)	15.696	< 0.001
Transverse diameter (mm)	1.065 (1.010–1.123)	5.341	0.021	1.076 (1.019–1.137)	6.950	0.008
LA area (4‐chamber view) (cm^2^)	1.058 (1.008–1.111)	5.259	0.022	1.086 (1.029–1.146)	8.920	0.003
LA area (2‐chamber view) (cm^2^)	1.211 (1.121–1.308)	23.824	< 0.001	1.307 (1.174–1.454)	24.005	< 0.001
LV function						
Left ventricular ejection fraction (LVEF, %)	0.663 (0.568–0.774)	27.265	< 0.001	—	—	—
E/e′ ratio	1.078 (0.988–1.177)	2.865	0.091	1.200 (1.062–1.357)	8.549	0.003
LV end‐diastolic volume (mL)	1.008 (0.996–1.021)	1.687	0.194	—	—	—
LV end‐systolic volume (mL)	1.042 (1.018–1.067)	11.999	0.001	1.064 (1.030–1.100)	13.794	< 0.001
Global longitudinal strain (%)	3.300 (1.955–5.572)	19.964	< 0.001	3.495 (2.023–6.036)	20.139	< 0.001
Pulmonary artery systolic pressure (mmHg)	1.052 (0.993–1.115)	2.962	0.085	1.092 (1.016–1.173)	5.659	0.017

*Note:* Logistic regression analyses were adjusted for age, body mass index, and hypertension and separately for LA and LV morphology and hemodynamics.

**Table 5:** Logistic regression analysis for left atrial (LA) function, reservoir, conduit, and booster pump.

Variable	Univariate	Wald	*p* value	Multivariate	Wald	*p* value
Phasic LA function						
Maximum LA volume (mL)	1.012 (0.999–1.024)	3.317	0.069	1.013 (0.999–1.028)	3.451	0.063
Minimum LA volume (mL)	1.026 (1.008–1.045)	7.915	0.005	1.029 (1.009–1.050)	7.834	0.005
Pre‐contraction LA volume (mL)	1.018 (1.001–1.034)	4.472	0.034	1.017 (0.998–1.036)	3.158	0.076
LA ejection fraction (%)	0.897 (0.854–0.942)	18.943	< 0.001	0.857 (0.798–0.921)	17.526	< 0.001
Reservoir						
Peak atrial strain at the end of ventricular systole (%)	0.852 (0.779–0.932)	12.248	< 0.001	0.850 (0.777–0.930)	12.596	< 0.001
LA emptying fraction (%)	0.955 (0.925–0.985)	8.277	0.004	0.946 (0.912–0.981)	9.073	0.003
LA expansion index (%)	0.973 (0.957–0.990)	9.467	0.002	0.968 (0.949–0.987)	11.061	0.001
Conduit						
Passive emptying volume (mL)	1.010 (0.973–1.047)	0.253	0.615	—	—	—
Passive emptying fraction (%)	1.009 (0.975–1.045)	0.266	0.606	1.050 (1.001–1.102)	4.072	0.044
Conduit volume	0.989 (0.966–1.011)	0.955	0.328	—	—	—
Passive emptying strain	0.971 (0.936–1.008)	2.363	0.124	0.927 (0.876–0.982)	6.789	0.009
Booster pump						
Peak atrial strain before atrial contraction (%)	0.983 (0.913–1.057)	0.217	0.642	—	—	—

*Note:* For the LA function, reservoir, conduit, and booster pump, logistic regression analyses were adjusted separately by age, body mass index, and hypertension.

Regarding LV function, higher E/e′ ratio (OR = 1.200, 95% CI: 1.062–1.357, *p* = 0.003), larger LV end‐systolic volume (OR = 1.064, 95% CI: 1.030–1.100, *p* < 0.001), and worse global longitudinal strain (OR = 3.495, 95% CI: 2.023–6.036, *p* < 0.001) were independently associated with AF recurrence (Table 5). PASP was also a significant predictor (OR = 1.092, 95% CI: 1.016–1.173, *p* = 0.017).

### 3.4 LA Function, Reservoir, Conduit, and Booster Pump

Among phasic LA function parameters, higher minimum LA volume was a strong predictor of AF recurrence (OR = 1.029, 95% CI: 1.009–1.050, *p* = 0.005), while LA ejection fraction was inversely associated with recurrence (OR = 0.857, 95% CI: 0.798–0.921, *p* < 0.001). Regarding LA reservoir function, lower PALS (OR = 0.850, 95% CI: 0.777–0.930, *p* < 0.001), reduced LA emptying fraction (OR = 0.946, 95% CI: 0.912–0.981, *p* = 0.003), and lower LA expansion index (OR = 0.968, 95% CI: 0.949–0.987, *p* = 0.001) were significantly associated with recurrence. For LA conduit function, the passive emptying fraction was an independent predictor (OR = 1.050, 95% CI: 1.001–1.102, *p* = 0.044), while passive emptying strain was inversely associated with recurrence (OR = 0.927, 95% CI: 0.876–0.982, *p* = 0.009). No booster pump parameters reached statistical significance in the multivariate model.

### 3.5 ROC Analysis

**Figure 3:**
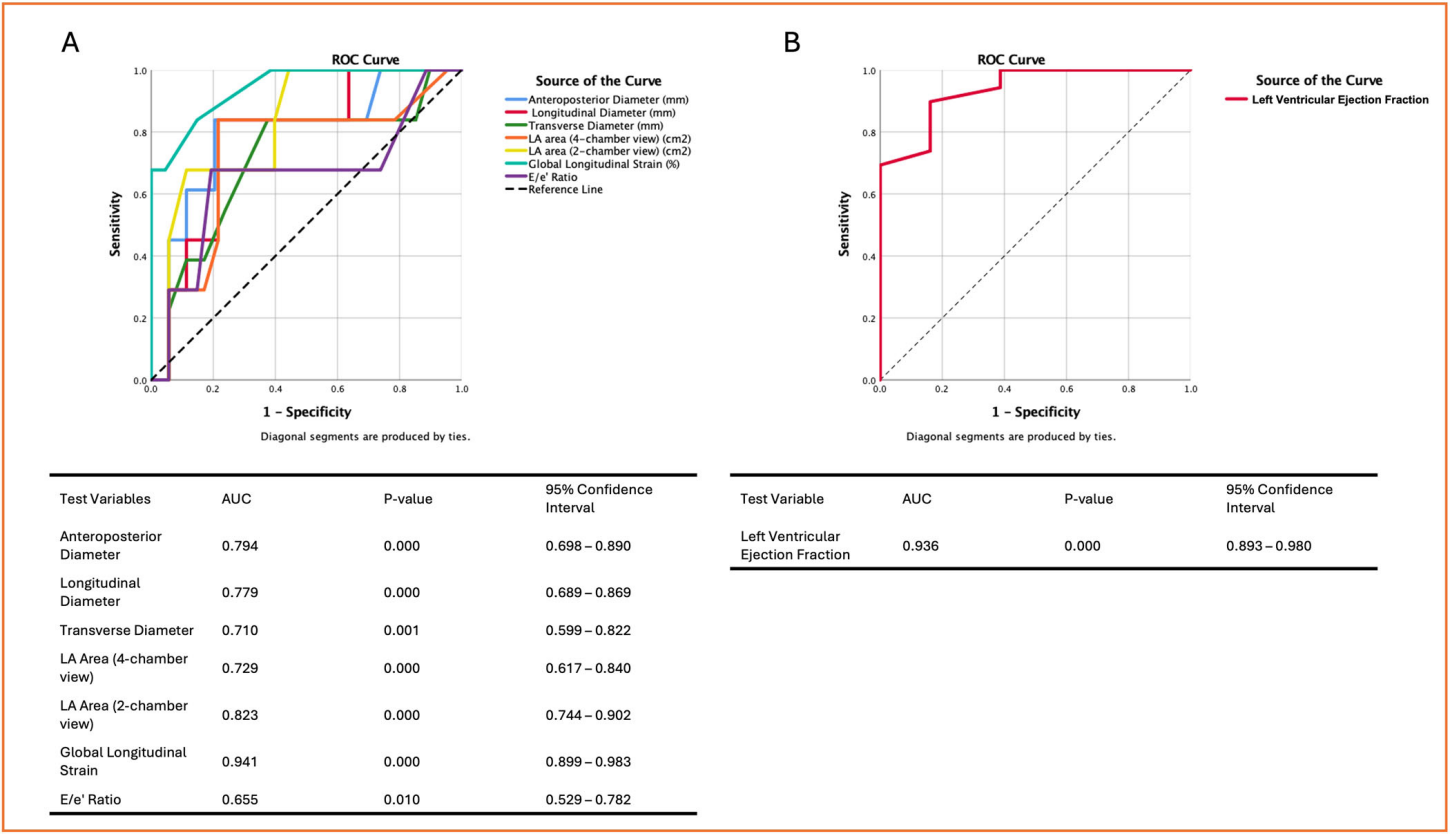
Receiver operating characteristic (ROC) curves for predicting atrial fibrillation recurrence. (A) ROC curves for left atrial (LA) morphological and functional parameters, including anteroposterior, longitudinal, and transverse diameters, LA area in four‐chamber and two‐chamber views, global longitudinal strain (GLS), and the E/e′ ratio. (B) The left ventricular ejection fraction (LVEF) ROC curve was analyzed separately, with the state variable set to 0 for recurrence.

**Figure 4:**
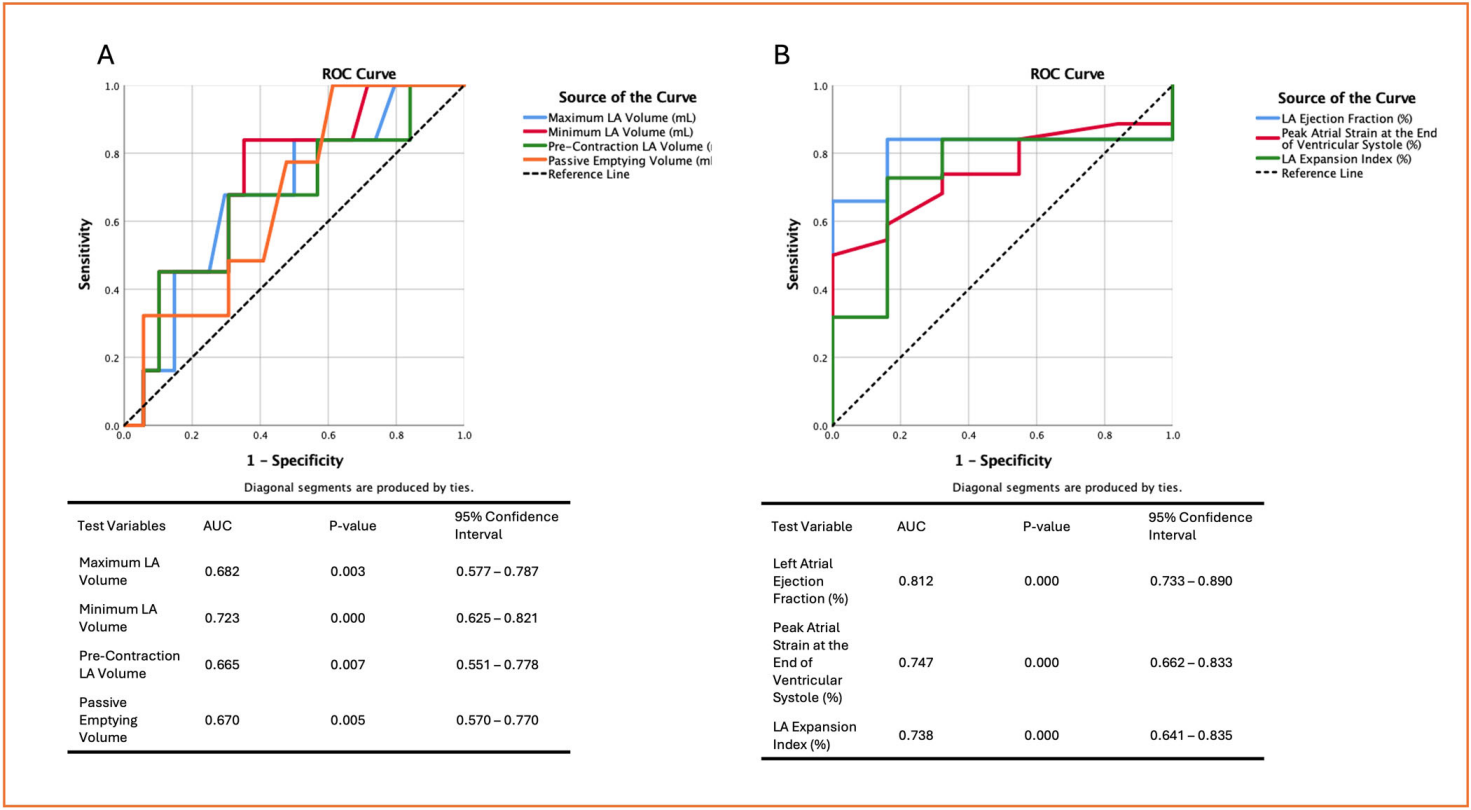
Receiver operating characteristic (ROC) curves for left atrial functional parameters predicting atrial fibrillation recurrence. (A) ROC curves for phasic left atrial (LA) volumes, including maximum LA volume (LAVmax), minimum LA volume (LAVmin), precontraction LA volume (Pre‐LAV), and passive emptying volume, with the state variable set to 1 for recurrence. (B) ROC curves for LA reservoir function parameters, including LA ejection fraction (LAEF), peak atrial strain at the end of ventricular systole (PALS), and LA expansion index, with the state variable set to 0 for recurrence.

The ROC curve analysis was performed to evaluate the discriminatory ability of echocardiographic parameters in predicting AF recurrence (Figures 3 and 4).

Multiple LA morphological and functional parameters demonstrated significant predictive value. Notably, GLS showed the highest discriminatory ability, with an AUC of 0.941 (95% CI: 0.899–0.983, *p* < 0.001) and an optimal cutoff of −13.5%, yielding a sensitivity of 83.9% and a specificity of 85.2%. The LA area in the two‐chamber view also exhibited excellent discriminatory performance (AUC: 0.823, 95% CI: 0.744–0.902, *p* < 0.001), with an optimal threshold of 23 cm² (sensitivity: 67.7%, specificity: 69.3%). Similarly, the LA area in the four‐chamber view had a good discriminatory ability (AUC: 0.729, 95% CI: 0.617–0.840, *p* < 0.001), with an optimal cutoff of 21.5 cm² (sensitivity: 83.9%, specificity: 55.7%). Regarding LA diameters, the anteroposterior diameter exhibited a good discriminatory ability (AUC: 0.794, 95% CI: 0.698–0.890, *p* < 0.001), with an optimal cutoff of 42.5 mm (sensitivity: 83.9%, specificity: 68.2%). The longitudinal diameter also showed good discriminatory power (AUC: 0.779, 95% CI: 0.689–0.869, *p* < 0.001), with an optimal cutoff of 59.5 mm (sensitivity: 83.9%, specificity: 73.9%). The transverse diameter had a moderate discriminatory ability (AUC: 0.710, 95% CI: 0.599–0.822, *p* = 0.001), with an optimal threshold of 43 mm (sensitivity: 83.9%, specificity: 53.4%). The E/e′ ratio showed lower discriminatory ability (AUC: 0.655, 95% CI: 0.529–0.782, *p* = 0.010), with a best cutoff 10.5, achieving a sensitivity of 67.7% and specificity of 56.8%. The LVEF was analyzed separately as a predictor of recurrence, with an AUC of 0.936 (95% CI: 0.893–0.980, *p* < 0.001). The optimal cutoff for LVEF was identified at 47%, balancing sensitivity (89.8%) and specificity (83.9%).

Figure 4A illustrates the ROC curves for LA phasic volumes predicting AF recurrence. Among these parameters, LAVmin demonstrated the highest predictive ability, with an AUC of 0.723 (95% CI: 0.625–0.821, *p* < 0.001). The optimal cutoff for LAVmin was 39.5 mL, achieving 67.7% sensitivity and 64.8% specificity. Similarly, LAVmax exhibited a moderate discriminatory ability (AUC: 0.682, 95% CI: 0.577–0.787, *p* = 0.003), with a cutoff of 42.5 mL (sensitivity: 83.9%, specificity: 73.9%). The Pre‐LAV also had moderate predictive power (AUC: 0.665, *p* = 0.007), with a cutoff of 40.5 mL(sensitivity: 67.7%, specificity: 56.8%). Additionally, passive emptying volume showed a similar trend (AUC: 0.670, *p* = 0.005), with an optimal cutoff of 7.5 mL, yielding 77.4% sensitivity and 52.3% specificity.

Figure 4B evaluates the reservoir function of the LA, where LAEF had the highest discriminatory ability, with an AUC of 0.812 (95% CI: 0.733–0.890, *p* < 0.001). The best cutoff for LAEF was 27.5%, with 84.1% sensitivity and 83.9% specificity, making it a strong predictor of AF recurrence. The peak atrial strain at the end of ventricular systole exhibited a good predictive value (AUC: 0.747, 95% CI: 0.662–0.833, *p* < 0.001), with an optimal threshold of 9.5% (sensitivity: 68.2%, specificity: 67.7%). Similarly, the LA expansion index demonstrated good discriminatory ability (AUC: 0.738, *p* < 0.001), with a cutoff of 47.5%(sensitivity: 65.9%, specificity: 83.9%).

## 4 Discussion

AF recurrence after PVI remains a significant clinical challenge, with nearly one‐fourth of patients experiencing arrhythmia recurrence despite ablation therapy [3, 10, 14]. In this study, we comprehensively evaluated LA morphology and function using 3D echocardiography and strain analysis to identify predictors of AF recurrence. Our findings highlight the pivotal role of LA structural remodeling, impaired reservoir function, and increased stiffness in predicting recurrence risk. Notably, larger LA dimensions emerged as independent predictors of recurrence, particularly in the two‐chamber view, along with reduced PALS and LAEF. Furthermore, impaired GLS and increased LV end‐systolic volume contributed to recurrence risk, underscoring the interplay between atrial and ventricular dysfunction in AF pathophysiology. These results emphasize the need for a multimodal approach to risk stratification in patients undergoing PVI, integrating advanced echocardiographic parameters to refine patient selection and optimize long‐term outcomes [5, 15, 16, 17, 18, 19, 20, 21].

Impaired LA and LV morphology and hemodynamics were associated with recurrence after PVI. Our study identified anteroposterior diameter and LA area in the four‐ and two‐chamber views as strong predictors of AF recurrence. These findings are consistent with the group of Montserrat et al., where an anteroposterior diameter > 40 mm was associated with a 1.8‐fold increased risk of AF recurrence [12]. Similarly, Bao et al. found that an LA area > 22 cm^2^ significantly predicted recurrence, aligning with our study's threshold of 23 cm^2^ [20]. Moreover, Sohns et al. further demonstrated that each 5 mm increase in anteroposterior diameter raised the recurrence risk by 22%, reinforcing the role of LA enlargement in AF persistence [1]. These findings highlight that LA dilation, particularly in the anteroposterior dimension and chamber area, is critical in arrhythmogenic remodeling and recurrence risk after PVI [2, 3, 4, 10, 20].

Regarding LV function, our results showed that lower and higher E/e′ ratios were significantly associated with AF recurrence, consistent with the previous report that each 5% reduction in LVEF increased the risk of recurrence by 20% [1, 16]. Additionally, it has been reported that patients with LVEF < 50% had a 2.3‐fold increased recurrence risk, and each unit increase in E/e′ ratio was associated with a 15% increased risk [4]. Kim et al. highlighted that GLS > ‐14% was an independent predictor of recurrence, which is in agreement with our study, where GLS > ‐13.5% had the highest discriminatory ability (AUC: 0.941, *p* < 0.001) [4]. Our study supports the growing evidence that LA structural remodeling and LV hemodynamic dysfunction are crucial in post‐PVI outcomes [2, 4, 10]. These findings could partially suggest that advanced echocardiographic assessment, incorporating strain imaging and volumetric analysis, may enhance risk stratification for AF recurrence.

Conversely, LA functional parameters, particularly LAEF, PALS, and the LA expansion index, were significant predictors of AF recurrence. The present results align with Montserrat et al., where LA volume > 50 mL/m^2^ was associated with a higher AF recurrence risk, and each 10 mL/m^2^ increase in LA volume was linked to a 17% higher likelihood of AF recurrence [12]. Similarly, another study found that patients with LA volume > 48 mL/m² had a 1.5‐fold increased risk of AF recurrence after ablation [20].

Additionally, previous data showed the role of LA strain parameters in predicting arrhythmia recurrence, demonstrating that each 1% reduction in PALS increased the risk of recurrence by 10% [3]. This aligns with our findings, where PALS < 10% (OR = 0.850, 95% CI: 0.777–0.930) was significantly associated with recurrence. The strong association between reduced LA reservoir strain and recurrence risk has been confirmed in multiple studies [5, 18]. It has been reported that a PALS cutoff of 9.7% (AUC = 0.740, sensitivity = 71%, specificity = 74%) was predictive of recurrence [18], closely matching our optimal threshold of 9.5% (AUC = 0.747, sensitivity = 68.2%, specificity = 67.7%). Moreover, another study found that a PALS cutoff of 11% was associated with a 2.4‐fold increased risk of AF recurrence, which is consistent with our findings where lower PALS significantly predicted recurrence (OR = 0.850, *p* < 0.001) [5].

The LA conduit function was also associated with higher recurrence. However, passive emptying strain was inversely associated with recurrence (OR = 0.927, 95% CI: 0.876–0.982, *p* = 0.009), suggesting impaired conduit function may contribute to abnormal atrial hemodynamics. This finding is supported by Reddy et al., who demonstrated that reduced LA conduit strain is a hallmark of left atrial dysfunction in heart failure with preserved ejection fraction [21].

Notably, LA booster pump function parameters did not reach statistical significance in the multivariate model. This contrasts with previous findings, which identified the late diastolic strain rate as a strong predictor of arrhythmia recurrence following a second ablation [12, 13]. The discrepancy may be attributed to differences in patient populations, procedural techniques, and echocardiographic methodologies.

Atrial mechanical stunning following cardioversion, particularly in patients with persistent atrial fibrillation, is a well‐documented phenomenon characterized by a transient impairment of atrial contractile function despite the restoration of sinus rhythm. This dysfunction has been observed with all modes of cardioversion (including electrical, pharmacologic, and spontaneous) [22, 23]. It is believed to result from the underlying atrial arrhythmia rather than the conversion method. Atrial stunning peaks immediately after cardioversion and generally resolves within minutes to 6 weeks, depending on the duration of prior AF, atrial size, and structural remodeling [22]. In our study, echocardiographic evaluation was performed 6 months postablation, where any residual stunning is unlikely to persist. Nevertheless, we acknowledge that in patients who remained in AF or recently converted at the time of imaging, some residual mechanical dysfunction cannot be entirely excluded, representing a theoretical limitation in the interpretation of atrial functional parameters.

A key strength of this study is the comprehensive echocardiographic evaluation, which includes 3D volumetric and strain‐based analyses, allowing for a detailed assessment of LA structure and function. The study benefits from a well‐defined prospective cohort and rigorous echocardiographic standardization, enhancing the reliability of the findings. Additionally, employing multivariate analysis and ROC curves strengthens the clinical applicability of our predictive model for AF recurrence after PVI.

However, this study has several limitations. First, it is a single‐center study with a moderate sample size, which may limit external validity. Second, while echocardiographic parameters were rigorously assessed, cardiac MRI (the gold standard for LA fibrosis quantification) was omitted, potentially limiting the mechanistic understanding of substrate remodeling. Third, the follow‐up duration was 6 months, which may not fully capture long‐term recurrence patterns. Future studies with larger populations, multimodal imaging, and extended follow‐up are needed to validate and expand on these findings. Fourth, standardized pre‐ablation 3D echocardiographic data are absent across the full cohort. Although some patients underwent echocardiography before pulmonary vein isolation, the imaging protocols were heterogeneous and uniformly available. As a result, we decided to restrict our analysis to the 6‐month postablation studies, which were obtained under consistent conditions and protocols. This limited our ability to analyze the dynamic changes in atrial structure and function over time.

This study highlights the crucial role of LA structural remodeling, impaired reservoir function, and LV dysfunction in predicting AF recurrence after PVI. Larger LA dimensions, particularly in the anteroposterior and two‐chamber areas, were independently associated with recurrence, while reduced PALS, LAEF, and GLS emerged as key functional predictors. These findings reinforce the need for a multimodal echocardiographic approach to enhance risk stratification and optimize patient selection for PVI. Integrating strain‐based and volumetric analysis into routine clinical practice could help refine postablation surveillance and individualized treatment strategies, ultimately improving long‐term rhythm outcomes in AF patients.

## Ethics Statement

This study was conducted using the principles outlined in the Declaration of Helsinki and was approved by the *Centro Medico Nacional 20 de Noviembre* Institutional Ethics Committee (05‐217‐2024).

## Consent

All participants provided written informed consent before inclusion in the study.

## Conflicts of Interest

The authors declare no conflicts of interest.

## Data Availability

The data that support the findings of this study are available from the corresponding author upon reasonable request. The data sets generated and analyzed during the current study are available from the corresponding author upon reasonable request.
